# Core Hunter 3: flexible core subset selection

**DOI:** 10.1186/s12859-018-2209-z

**Published:** 2018-05-31

**Authors:** Herman De Beukelaer, Guy F Davenport, Veerle Fack

**Affiliations:** 1 Department of Applied Mathematics, Computer Science and Statistics, Ghent University, Krijgslaan 281 S9, Gent, 9000 Belgium; 2 New Zealand Institute for Plant & Food Research Limited, 412 No1 Rd RD2, Te Puke, New Zealand

**Keywords:** Core collections, Multi-objective, Local search heuristics

## Abstract

**Background:**

Core collections provide genebank curators and plant breeders a way to reduce size of their collections and populations, while minimizing impact on genetic diversity and allele frequency. Many methods have been proposed to generate core collections, often using distance metrics to quantify the similarity of two accessions, based on genetic marker data or phenotypic traits. Core Hunter is a multi-purpose core subset selection tool that uses local search algorithms to generate subsets relying on one or more metrics, including several distance metrics and allelic richness.

**Results:**

In version 3 of Core Hunter (CH3) we have incorporated two new, improved methods for summarizing distances to quantify diversity or representativeness of the core collection. A comparison of CH3 and Core Hunter 2 (CH2) showed that these new metrics can be effectively optimized with less complex algorithms, as compared to those used in CH2. CH3 is more effective at maximizing the improved diversity metric than CH2, still ensures a high average and minimum distance, and is faster for large datasets. Using CH3, a simple stochastic hill-climber is able to find highly diverse core collections, and the more advanced parallel tempering algorithm further increases the quality of the core and further reduces variability across independent samples. We also evaluate the ability of CH3 to simultaneously maximize diversity, and either representativeness or allelic richness, and compare the results with those of the GDOpt and SimEli methods. CH3 can sample equally representative cores as GDOpt, which was specifically designed for this purpose, and is able to construct cores that are simultaneously more diverse, and either are more representative or have higher allelic richness, than those obtained by SimEli.

**Conclusions:**

In version 3, Core Hunter has been updated to include two new core subset selection metrics that construct cores for representativeness or diversity, with improved performance. It combines and outperforms the strengths of other methods, as it (simultaneously) optimizes a variety of metrics. In addition, CH3 is an improvement over CH2, with the option to use genetic marker data or phenotypic traits, or both, and improved speed. Core Hunter 3 is freely available on http://www.corehunter.org.

## Background

Genebanks were established by national or international breeding, or conservation programs with the goal to safeguard genetic diversity for future use. Many breeding programs have established genebanks as a resource for new variation in the crops they breed, allowing them to react to changing environments and emerging biotic and abiotic stresses. Accessions are often divided between active (or working) and base collections. Examples of active collections include seed stores or live plants that can be accessed quickly by plant breeders and researchers through germination or clonal propagation. In contrast, accessions in base collections are held in long-term storage, such as cryopreservation, and require some time for regeneration and propagation before being made available.

During the last few decades the collections stored in genebanks have grown enormously, and cost of maintaining viable germplasm within genebanks has increased. Genebank curators must make decisions about which accessions to maintain in the active collection versus the base collection, and may even consider not maintaining an accession at all. The concept of a core collection was introduced to help with these decisions, and is defined as subset of the complete collection which most represents the diversity of the entire collection with minimum redundancy [1]. Genebank curators can use core collections to define the active collection over the base collection. Core collections can also be used to aid researchers and plant breeders in the choice of starting material. For example, the potential for use of core collections has been shown for association studies [2, 3].

A variety of measures have been used to evaluate core collections based on genetic marker data or phenotypic traits, including pairwise distances and allelic richness. The choice of the most appropriate evaluation measure depends on the purpose of the core collection [4]. Sometimes core collections are sampled based on a combination of both genotypes and phenotypes [5–7]. Many methods have been proposed to sample high quality core collections according to the measure(s) of interest. The first methods were stratified sampling techniques that cluster the accessions, based on distance matrices calculated from their allele scores or phenotypic trait values, and then select several accessions from each cluster using a certain allocation method. Brown suggested to randomly select either a constant (C) number of accessions per cluster, or a number proportional (P) to the size or logarithm (L) of the size of the cluster, and argued that the L-method is preferred [8]. It was later shown that more diverse cores are obtained when the number of included accessions is proportional to the within-cluster diversity [9].

Another allocation method, the M-method, maximizes the probability to retain all observed alleles in order to construct cores with high allelic richness [10]. This idea led to the development of the MSTRAT software, which implements a generalized M-method that directly samples from the entire collection to maximize allelic richness with a simple hill-climbing algorithm [11]. Other heuristics work by repeatedly removing one of the two most similar accessions from the collection until the desired core size is obtained, either randomly (least distance stepwise sampling [12]), or using a specific elimination criterion maximizing the distance to the remaining accessions or expected heterozygosity of the reduced collection (SimEli) [13]. The genetic distance optimization strategy (GDOpt) was designed to construct highly representative cores, in which each accession from the entire collection is represented by a similar core entry [14]. GDOpt partitions the data around a number of identified medoids, which are then selected as the core entries. Methods for variable size core sampling have also been developed. PowerCore minimizes the size of the core, while covering all observed marker alleles and/or trait values [15]. GenoCore was developed for the same purpose, and specifically tailored to high-density marker datasets [16]. The genetic distance sampling strategy constructs cores with a given minimum distance between selected accessions by repeatedly including a random accession and removing all others within a certain sampling radius [17].

Core Hunter was designed to meet the variety of criteria used to evaluate core collections for different purposes, and supports optimization of several of these metrics, using flexible local search algorithms [18]. Core Hunter can construct core collections for specific applications, and combines multiple objectives to bring the different perspectives closer together, for example by simultaneously maximizing genetic dissimilarity and allelic richness. Although Core Hunter is mainly focused at fixed size core subset selection, version 1 and 2 allowed to specify a minimum and maximum size and preferred smaller cores with the same value. Core Hunter was shown to outperform stratified sampling strategies, MSTRAT and PowerCore.

It has been assumed that, to obtain a diverse core, the average distance between its entries should be maximized [9, 18]. However, a high entry-to-entry distance does not guarantee that selected accessions are sufficiently different, and it is known that maximizing this criterion overrepresents extreme values [4, 19]. Core Hunter 2 (CH2) deals with this issue by also maximizing the minimum distance between selected accessions [19]. Although average distance and allelic richness can be effectively optimized using simple and fast local search algorithms, such as a stochastic hill-climber, a more complex and slower *mixed replica search* (MixRep) was required to maximize minimum distance in the Core Hunter framework. The MixRep algorithm runs multiple types of stochastic local searches in parallel, as well as a constructive algorithm (LR) that starts from an empty selection that is iteratively extended. In case an active search is unable to find any further improvements, it is terminated and replaced with a new local search engine starting from a selection that is obtained by combining two previously found high-quality selections, in an attempt to further explore other interesting regions of the search space, as in a genetic algorithm [20].

Another approach to maximize diversity, while at the same time avoiding inclusion of too similar accessions at the extremes of the collection, is to maximize the average distance between each entry and the closest other entry in the core, as proposed by Odong et al. [4]. The SimEli algorithm was shown to outperform Core Hunter 2 in terms of this new entry-to-nearest-entry (E-NE) metric. Alternatively, one may desire to optimally represent the individual accessions, instead of the whole range of diversity. In such case, Odong et al. reccomend to minimize the average distance between each accession in the full collection and the most similar accession contained in the core. The GDOpt strategy was specifically developed to minimize this accession-to-nearest-entry (A-NE) metric, and shown to outperform both Core Hunter 2 and SimEli for this purpose [13, 14].

We introduce Core Hunter 3 (CH3), which incorporates the two improved methods for summarizing distances, entry-to-nearest-entry (E-NE) and accession-to-nearest-entry (A-NE), proposed by Odong et al. [4]. CH3 attempts to find the maximum entry-to-nearest-entry distance to obtain diverse cores, whereas accession-to-nearest-entry distance is minimized to represent as much as possible all accessions from the entire collection. More specifically, CH3 can sample fixed size cores based on molecular marker data, phenotypic traits, a precomputed distance matrix, or a combination of these. The distance matrix can be generated using an appropriate measure, such as Modified Roger’s distance for genotypes [21] or Gower’s distance for phenotypes [22]. As in previous versions, Core Hunter 3 can also maximize allelic richness, as well as a combination of multiple metrics. In particular, we assess whether the new distance-based E-NE and A-NE metrics can be effectively optimized using fast local search algorithms, and whether maximizing E-NE indirectly also yields a high minimum distance, without the need for a more complex algorithm. Furthermore, we assess the ability of Core Hunter 3 to simultaneously maximize E-NE and A-NE, or E-NE and allelic richness, and compare the results with those obtained with Core Hunter 2, GDOpt, and SimEli, for three marker datasets with different allelic composition and varying size, and one phenotypic trait dataset. Core Hunter 3 is available as an R package *corehunter* on CRAN and as an open source project on GitHub. A prototype graphical user interface is also available. See http://www.corehunter.org for more information.

## Methods

### Core selection problem

Given a collection $\mathcal {A}$ that contains *n* accessions, and a desired core size 1<*k*<*n*, the feasible solution space of possible core subsets is defined as

$$ \Omega = \left\{ \mathcal{C} \mid \mathcal{C} \subset \mathcal{A} \land \left\vert\mathcal{C}\right\vert = k \right\} $$

where $\left \vert \mathcal {C}\right \vert $ denotes the size of the subset. The core selection problem then consists of finding an optimal subset $\mathcal {C^{*}} \in \Omega $ that maximizes a certain evaluation measure $F(\mathcal {C}): \Omega \rightarrow \mathbb {R}$, i.e.

$$ \mathcal{C^{*}} = \underset{\mathcal{C} \in \Omega}{\text{argmax}}\,F(\mathcal{C}). $$

In case the evaluation measure $F(\mathcal {C})$ is intended to be minimized, this can be achieved by maximizing $-F(\mathcal {C})$.

### Evaluation measures

Core Hunter 3 includes various evaluation measures that can be selected as optimization objectives, including but not limited to those described below. We refer to the website http://www.corehunter.org for an overview of all provided measures.

#### Distance measures

We used the Modified Roger’s distance [18, 21] to assess the dissimilarity of accessions based on genetic marker data. For phenotypic traits we used Gower’s distance [22] which simultaneously takes into account qualitative and quantitative traits. Pairwise distances are aggregated as follows to evaluate the diversity or representativeness of the core [4]:

- Entry-to-nearest-entry (E-NE): the average distance between each selected accession and the closest other core entry. This criterion can be maximized to construct highly diverse cores in which all accessions are maximally different.
- Accession-to-nearest-entry (A-NE): the mean distance between each accession from the entire collection and the most similar core entry, including itself in case the accession has been selected. Minimizing this criterion yields cores that maximally represent all individual accessions.

When comparing CH3 with CH2 we also evaluated the minimum distance (DMIN) between selected accessions, but this is not an objective that can be directly optimized by CH3, for reasons explained in the discussion. A detailed description and comparison of the E-NE and A-NE metrics are provided in [4].

#### Allelic richness

To evaluate the allelic richness of cores sampled based on genetic marker data, we used the average expected heterozygosity (HE) per locus [18, 23], calculated as

$$ 0 \le HE = \frac{1}{L}\sum_{l=1}^{L}{\left(1 - \sum_{a=1}^{n_{l}}{\hat{p}^{2}_{la}}\right)} \le 1 $$

where *L* is the number of markers (loci), *n*_*l*_ is the number of observed alleles at the *l*th locus, and $\hat {p}^{2}_{la}$ is the frequency of the *a*th allele at the *l*th locus in the selected core collection.

#### Weighted index and normalization

As in previous versions, Core Hunter can simultaneously optimize *k* measures by maximizing a weighted index

$$ F(c) = \sum_{i=1}^{k}{\alpha_{i} F_{i}(c)} $$

where *F*_*i*_ is the *i*th included evaluation measure and 0<*α*_*i*_<1 is the weight assigned to this objective, with $\sum _{i=1}^{k} \alpha _{i} = 1$. In case of a measure *F*_*i*_ that is to be minimized, such as A-NE, it is transformed into a maximization objective $F^{\prime }_{i} = -F_{i}$ when it is included in the weighted index. The individual measures are automatically normalized to [0,1], following the Pareto minimum based upper-lower-bound approach as described in [24], to ensure a fair balance between the included objectives, independent of their original range. More information about this normalization is provided in the documentation of the R package.

### Core sampling algorithms

We evaluate the performance of three general purpose selection heuristics to optimize the chosen evaluation measure or weighted index for a fixed core size: random descent, parallel tempering, and a genetic algorithm. Based on the findings in this study, only the former two were included in Core Hunter 3, which defaults to the parallel tempering algorithm, but also provides a fast mode in which the random descent algorithm is applied. Note that these two stochastic local search algorithms were also available in CH2, although they were not used by default. The search algorithms are executed until either an absolute runtime limit has been exceeded, or no further improvements were obtained during a certain amount of time.

#### Random descent

This basic local search outlined in Algorithm 1 starts with a random selection of the desired size and then iteratively tries to improve its quality by slightly modifying the core. The obtained similar selection, referred to as a *neighbour* of the current selection, is accepted if and only if it has a higher objective function value according to the chosen evaluation measure. Otherwise, another move is tried from the current selection. Core Hunter uses a *single-swap neighbourhood*, i.e. considers all neighbours that can be obtained from the current selection by replacing one selected accession with a currently unselected accession.


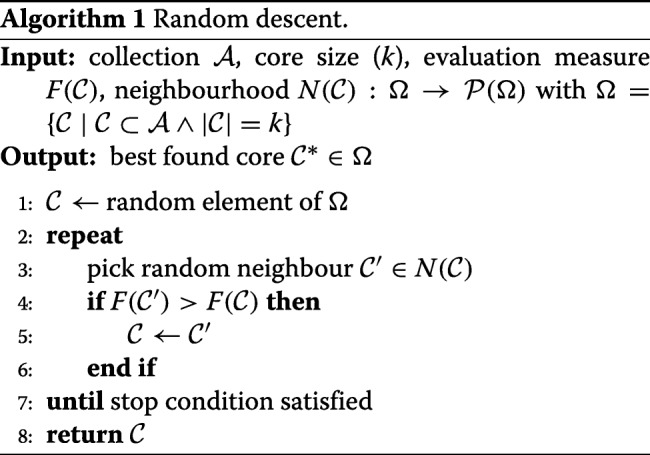


#### Parallel tempering

Algorithm 2 describes the more advanced *parallel tempering* method [18], also referred to as replica exchange Monte Carlo (REMC), which consists of multiple cooperating local searches that are executed in parallel. Each search performs the same procedure as random descent, but may also accept inferior modifications to be able to escape from local optima, i.e. to further improve the current selection even if none of the considered neighbours has a better score. For this purpose, the search replicates are assigned fixed, increasing temperatures, equally spread in a given range. A higher temperature leads to a higher probability to accept inferior modifications, similar to the frequently used *simulated annealing* algorithm [25]. The acceptance function is commonly defined as

$$p(\Delta,t) = \left\{ \begin{array}{ll} 1 & \text{if } \Delta > 0 \\ e^{\Delta/t} & \text{else} \end{array}\right. $$

where $\Delta = F(\mathcal {C}^{\prime }_{i}) - F(\mathcal {C}_{i})$ and *t* is the temperature of the replica. This acceptance function ensures that neighbours with a better score are always accepted, whereas inferior neighbours are accepted at a probability that exponentially decreases as the solution gets poorer or as the temperature is decreased. In addition, searches with similar temperature periodically exchange their current selection, which has the effect to push the most promising solutions towards the coolest searches to promote convergence towards a common solution, and the worst solutions towards the hottest searches allowing them to escape from local optima. The probability that replica *r* and *r*+1 will swap their current selection is commonly defined as

$$q\left(\Delta_{r}, t_{r}, t_{r+1}\right) = \left\{ \begin{array}{ll} 1 & \text{if }\Delta > 0 \\ e^{\left(\frac{1}{t_{r}} - \frac{1}{t_{r+1}}\right)\Delta} & \text{else} \end{array}\right. $$

with $\Delta _{r} = F\left (\mathcal {C}_{r+1}\right) - F(\mathcal {C}_{r})$. As such, if the current selection of replica *r*+1 has a better objective function value than that of the *r*th replica, these are always swapped. In addition, similar to the probabilistic acceptance of inferior neighbours, swaps that push solutions in the opposite direction may also be performed—yet with a probability that decreases for a larger difference in objective function value and replica temperature. The parallel tempering algorithm implemented in Core Hunter 3 consists of *p*=10 searches with a temperature range of [10^−8^,10^−4^], and uses the same single-swap neighbourhood as the random descent method described above. The number of replica steps per iteration is fixed to *q*=500, and the default acceptance and swap functions are applied.


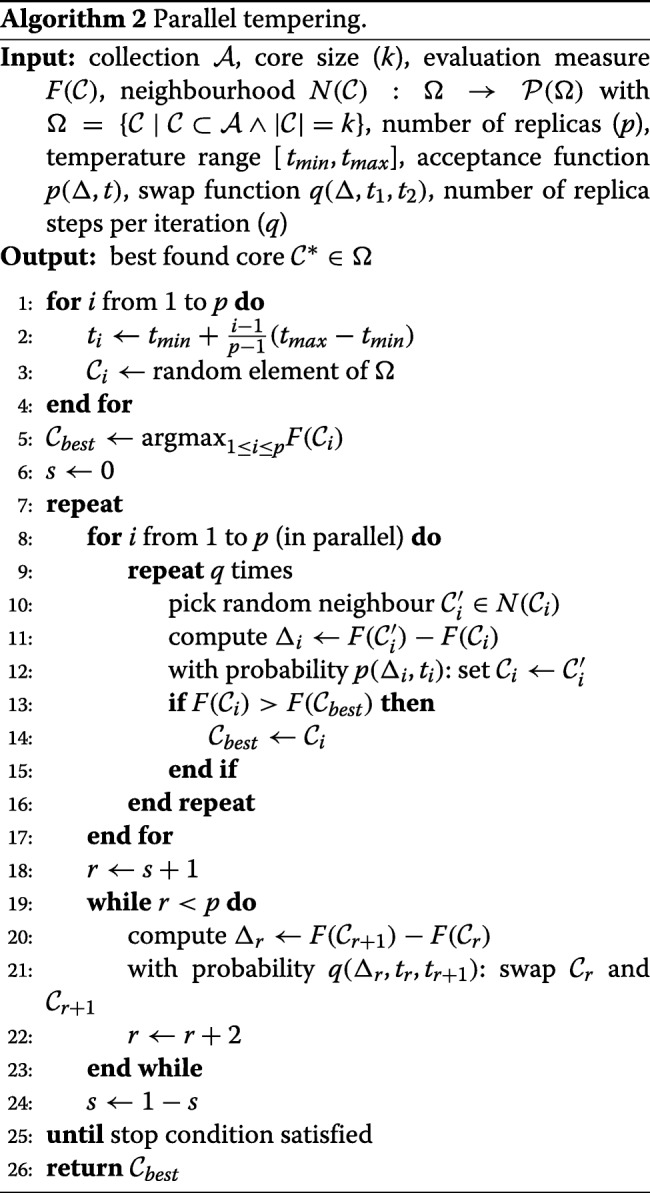


#### Genetic algorithm

To assess the potential improvement of a global optimization engine over a local search we also applied the genetic algorithm [20] outlined in Algorithm 3. Here, a population of initially randomly generated solutions (cores) is maintained. In every step, new *child* solutions are produced by combining two randomly chosen *parent* solutions (crossover), followed by one or more swaps of accessions (mutation) between the unselected and the selected subset. These children are added to the population, and certain solutions are discarded to simulate survival of the fittest individuals in natural evolution. For our experiments we used a population size of *p* = 25 and generated *c* = 5 children in each step (in parallel). We applied the following operators:

- **Selection** (Select).We randomly picked five candidates from the current population, from which the one with the highest objective function value was chosen as a parent (tournament selection).
- **Crossover** (Cross). A child was created from two parents by repeatedly adding an arbitrary accession that is selected in either parent solution (at random with equal probability) until the desired core size was obtained.
- **Mutation** (Mutate). As mutation operator we applied the random descent heuristic described above, starting from the given solution, until no improvement was found in the last 5000 steps.
- **Survival** (Survive). We applied a roulette selection to discard five solutions in each step, so that the population size remained fixed over all generations. A solution $\mathcal {C}$ was assigned a weight of $1/F(\mathcal {C})$ meaning that the probability that it is discarded is inversely proportional to its fitness.

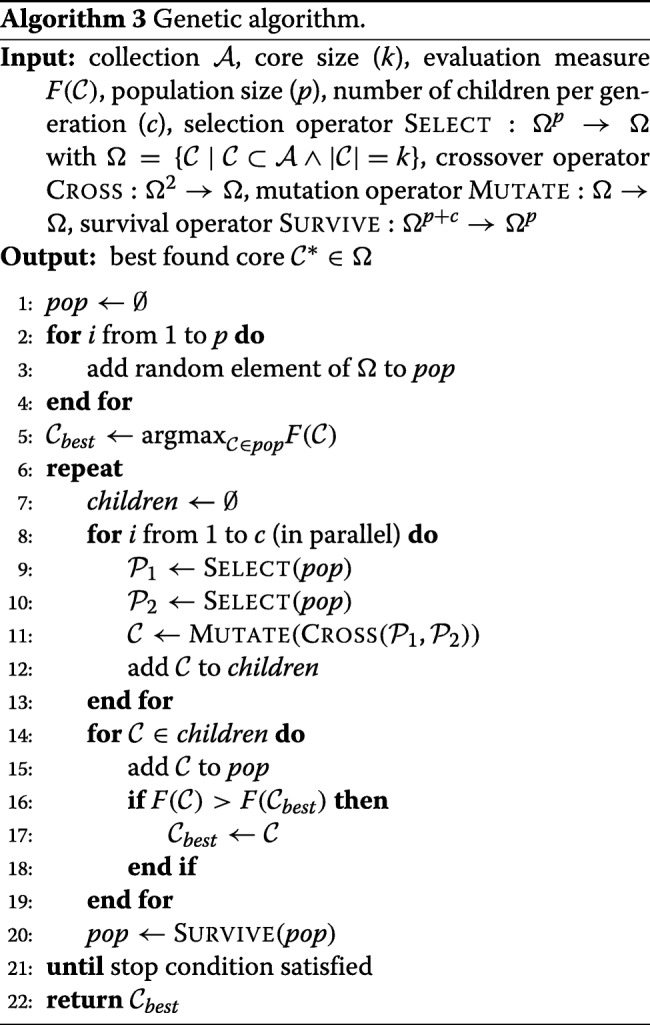


### Comparison with GDOpt and SimEli

For the GDOpt selection strategy [14], we used the k-medoids algorithm of Kaufman and Rousseuw [26] through the R function pam, to identify a representative core collection. The number of clusters was chosen equal to the desired core size and the returned medoids were selected as core accessions. We also implemented SimEli in R, considering both elimination criteria suggested in [13]. In each step, one of the two most similar accessions was eliminated, maximizing either the average distance to the remaining accessions (SimEli-A-RA) or the expected heterozygosity of the reduced collection (SimEli-HE), until the desired core size was obtained. The source code for these implementations is available on GitHub (https://github.com/corehunter/corehunter3-papercorehunter/corehunter3-paper).

### Datasets

We used four datasets of varying size and composition to compare the performance of different core sampling algorithms:

1. *Rice data*: 1000 accessions for which 39 phenotypic traits were recorded, including 28 qualitative and 11 quantitative traits. Available from the PowerCore project [15] and previously used to assess the performance of several other core sampling algorithms, including SimEli [13].
2. *Coconut data*: 1014 accessions characterized using 30 crop-specific SSR markers. Used in multiple previous core selection studies [4, 13, 14].
3. *Maize data*: 1250 accessions characterized with 1117 SNP markers. Distributed as part of the R package *synbreedData* [27].
4. *Pea data*: 4428 accessions characterized by 17 RBIP markers [28, 29]. Previously used to compare the performance of Core Hunter 2 with other core sampling algorithms for large datasets [19].

All cores sampled in the performed experiments comprised 20% of the entire collection for the rice, coconut and maize datasets, and 10% for the large pea dataset.

### Implementation and hardware

Core Hunter 3 has been reimplemented in Java 8, using the JAMES framework (v1.2) for discrete optimization with local search metaheuristics [30] and was executed from R through the package *corehunter* (https://cran.r-project.org/package=corehunter). GDOpt, SimEli, and all computational experiments were implemented in R v3.3.1 [31]. Note that the R function pam used in GDOpt calls a C function which performs the actual partitioning. Experiments were executed on a computing server with two 10-core Intel E5-2660v3 (2.6 GHz) CPUs and 128 GB RAM.

## Results

### Optimizing E-NE and A-NE with local searches

**Fig. 1:**
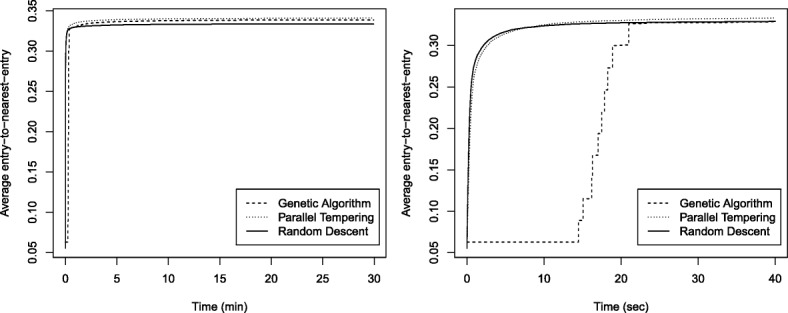
Convergence curves for pea dataset. These curves show the E-NE value of the best found solution at each point in time during execution of random descent, parallel tempering, and the genetic algorithm, averaged over 10 independent runs, for the large pea dataset. The left plot reports the progress during the entire run with a runtime of 30 min while the right plot is zoomed in on the first 40 s

We sampled 10 cores from each dataset using random descent, parallel tempering, and the described genetic algorithm, configured to maximize E-NE with a runtime limit of 30 min. Table 1 shows mean values and standard deviations of the obtained cores. The results indicate that parallel tempering yields the highest E-NE values, with the lowest variability across independent samples. Variability in solution quality is always at least one order of magnitude below that observed for random descent and the genetic algorithm. Still, variability is already quite low when using the basic random descent heuristic. Although the genetic algorithm also outperforms random descent, it is not as effective as parallel tempering. We performed a pairwise comparison of the results obtained with the three applied methods, for the four considered datasets, using a Wilcoxon rank-sum test [32]. The twelve resulting *p*-values were adjusted for multiple testing to control the family-wise error rate (FWER) using Holm’s method [33]. All differences were statistically significant at the *α*=0.05 confidence level, with adjusted *p*-values ranging from 0.00013 to 0.00049. Figure 1 displays convergence curves of the three applied algorithms, again averaged over 10 runs, for the large pea dataset. These plots confirm that all algorithms are able to iteratively improve an arbitrarily bad random selection to reach a high E-NE value. Again we see that parallel tempering yields the highest-quality cores (left). Moreover, this algorithm is almost as fast as the basic random descent heuristic (right). Both methods very quickly improve the initial random selection, and after less than 10 s, parallel tempering found a better solution than random descent, after which it keeps improving the quality of the core. In contrast, the genetic algorithm takes a slower start, catches up with random descent after 20 s, and then also further improves the selection—but not as effectively as parallel tempering. We performed these experiments only for the E-NE measure but assume that our findings also hold for A-NE due to the very similar composition of both criteria. All following CH3 results were obtained with the parallel tempering algorithm.

**Table 1:** Comparison of random descent, parallel tempering, and a genetic algorithm, when maximizing the entry-to-nearest-entry criterion (E-NE). Mean values and standard deviations are reported for 10 independently sampled core collections

	Rice	Coconut	Maize	Pea
Random descent	0.1500 ± 1.83e-04	0.5748 ± 5.22e-04	0.4332 ± 2.73e-04	0.3337 ± 1.70e-03
Parallel tempering	0.1508 ± 1.40e-15	0.5759 ± 2.12e-06	0.4359 ± 8.56e-05	0.3412 ± 1.46e-04
Genetic algorithm	0.1506 ± 1.12e-04	0.5755 ± 1.04e-04	0.4346 ± 3.45e-04	0.3386 ± 8.00e-04

### Comparison with Core Hunter 2

To assess whether maximizing E-NE indirectly also yields a high minimum distance (DMIN) between selected accessions, we compared the results of CH3 and CH2. We configured CH2 to maximize a weighted index including both average and minimum pairwise distance, with equal weight, and CH3 to maximize E-NE. Both algorithms were terminated when no improvement was found during the last 10 s. Table 2 reports average E-NE, DMIN, and execution time for 10 independent samples, obtained with both methods, and for each dataset except the rice collection, because CH2 cannot sample cores based on phenotypic traits. For all three datasets, CH3 yields higher E-NE and DMIN than CH2. However, a detailed inspection of the output generated by CH2 (not shown) revealed that the LR replica—one of the search replicas in the MixRep algorithm used by CH2—did not always complete before CH2 was terminated. This LR search is a constructive heuristic that starts with an empty selection and iteratively adds the two best accessions, i.e. those yielding the best possible score when added to the current selection. After each two additions, one accession is removed from the selection, again chosen to optimize the score of the remaining selection. This procedure is repeated until the desired core size has been reached. The LR replica was specifically included in CH2 to construct cores with high minimum distance [19]. Therefore, we repeated the CH2 experiments with an absolute runtime limit that was empirically determined per dataset to ensure that the LR replica terminated in each run (CH2L). Especially for the large pea dataset, significantly more time was needed in this configuration. Table 2 shows that CH2L is indeed able to construct cores with a much higher minimum distance than CH2, and also outperforms CH3 in terms of this measure. Although differences in minimum distance obtained with CH2L and CH3 are not larger than 4%, they are statistically significant for the coconut and maize datasets (*p*=0.000097), but not for the pea dataset (*p*=0.3064). Moreover, CH3 still yields significantly higher-quality core collections in terms of the E-NE criterion (*p*=0.000097), and is faster for large datasets.

**Table 2:** Comparison of Core Hunter 2 and 3

	E-NE	DMIN	Time (s)
*Coconut*			
CH2	0.552 ± 3.53e-2	0.501 ± 9.76e-2	27.6 ± 06.0
CH3	**0.576** ± 9.35e-5	0.540 ± 0.00e-0	37.5 ± 07.9
CH2L	0.569 ± 5.91e-4	**0.548** ± 0.00e-0	31.0 ± 00.1
*Maize*			
CH2	0.416 ± 1.52e-2	0.396 ± 2.46e-2	78.3 ± 10.6
CH3	**0.435** ± 2.70e-4	0.409 ± 3.05e-3	74.3 ± 26.5
CH2L	0.429 ± 5.00e-4	**0.415** ± 1.11e-3	78.6 ± 02.0
*Pea*			
CH2	0.219 ± 1.49e-3	0.000 ± 0.00e-0	85.6 ± 04.5
CH3	**0.338** ± 1.04e-3	0.287 ± 1.34e-2	154.1 ± 49.7
CH2L	0.325 ± 8.21e-4	**0.297** ± 0.00e-0	802.3 ± 00.8

CH2 maximizes a weighted index including average and minimum pairwise distance, with equal weight, while CH3 maximizes E-NE. Mean E-NE, DMIN, runtime and corresponding standard deviations are reported for 10 independent executions. The highest obtained E-NE and DMIN value per dataset is shown in bold. CH3 was terminated when no improvements were found during 10 s. For CH2, two alternatives were considered: (a) the same stop condition as for CH3 (CH2); and (b) an absolute runtime limit that was empirically determined per dataset to ensure that the LR replica of MixRep terminated in each run (CH2L)

### Comparison with GDOpt and SimEli

**Fig. 2:**
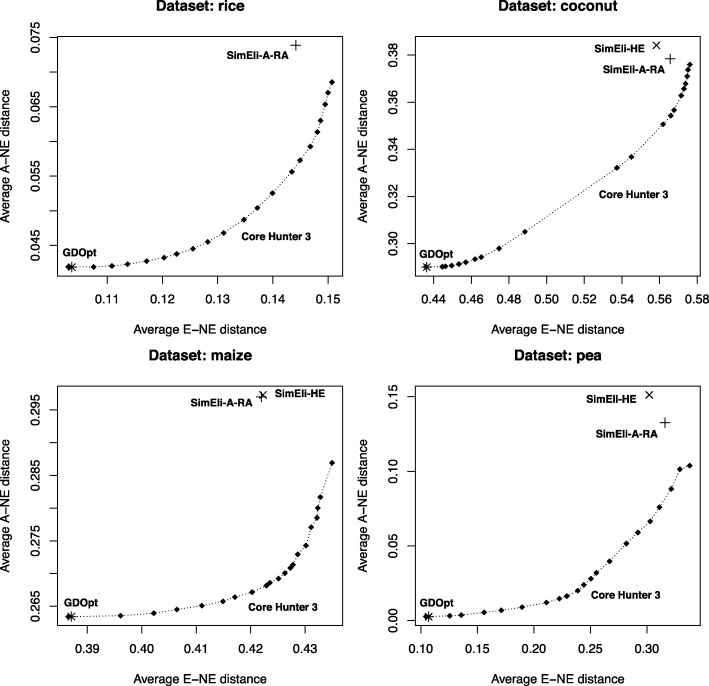
Simultaneous optimization of entry-to-nearest-entry (E-NE) and accession-to-nearest-entry (A-NE) distance. These Pareto front approximations for Core Hunter 3 were obtained by sampling cores with varying weights *α*_1_∈[ 0,1] and *α*_2_=1−*α*_1_ assigned to the E-NE and A-NE measures, respectively, with a step size of 0.05. The quality of the cores constructed by CH3 is compared with those obtained by GDOpt and SimEli, in terms of both objective functions. All reported values are averages of 10 independently sampled cores with the same settings

**Fig. 3:**
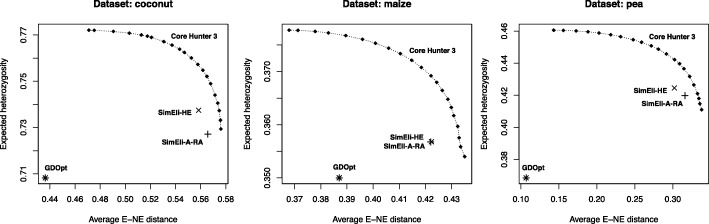
Simultaneous maximization of entry-to-nearest-entry distance (E-NE) and expected heterozygosity (HE). These Pareto front approximations for Core Hunter 3 were obtained by sampling cores with varying weights *α*_1_∈[ 0,1] and *α*_2_=1−*α*_1_ assigned to the E-NE and HE measures, respectively, with a step size of 0.05. The quality of the cores constructed by CH3 is compared with those obtained by GDOpt and SimEli, in terms of both objective functions. All reported values are averages of 10 independently sampled cores with the same settings. The rice dataset is excluded here because expected heterozygosity can only be evaluated for genotypic data

We approximated the Pareto front obtained by Core Hunter 3 when simultaneously optimizing E-NE, and either A-NE or HE, with varying weights *α*_1_∈[ 0,1] and *α*_2_=1−*α*_1_, respectively, and compared the results with those obtained by GDOpt and SimEli. Note that A-NE is minimized, while E-NE and HE are maximized. As before, CH3 was terminated when no improvement was found during 10 s. Figure 2 shows that GDOpt and CH3 are able to construct representative cores with low A-NE, which is not the case for SimEli. In fact, all cores sampled by SimEli have a worse A-NE value than those obtained by GDOpt and CH3, even when the latter is configured to maximize E-NE only. On the other hand, SimEli scores much better than GDOpt in terms of diversity (high E-NE). Still, Core Hunter 3 is able to find cores which simultaneously have a higher diversity and are more representative than those obtained with SimEli. For the maize dataset, SimEli-A-RA and SimEli-HE found cores of similar quality, while for the coconut and pea dataset SimEli-A-RA showed to be preferred in terms of both E-NE and A-NE. For the rice dataset, SimEli-HE was not included because expected heterozygosity can only be evaluated for genotypic data. Figure 3 shows that GDOpt yields cores with significantly lower HE than any of the other methods. SimEli performs better in this respect, especially SimEli-HE, but as before Core Hunter 3 is able to simultaneously improve over SimEli in terms of both objectives (E-NE and HE value).

Average execution times of GDOpt, SimEli and CH3 (configured to optimize E-NE, A-NE or HE) are reported in Table 3. Core Hunter 3 was slower than GDOpt and SimEli for the rice and coconut datasets. For the maize dataset CH3 was faster than GDOpt and SimEli-HE when maximizing HE or E-NE but slower when minimizing A-NE and always slower than SimEli-A-RA. Finally, for the pea dataset, CH3 was faster than both GDOpt and SimEli. Core Hunter 3 was also consistently faster when maximizing HE as compared to the configurations where E-NE or A-NE were optimized.

**Table 3:** Average execution times (seconds) of GDOpt, both SimEli implementations and CH3 for 10 independent samples from each dataset. Three configurations are considered for CH3: (a) maximize E-NE; (b) minimize A-NE; and (c) maximize HE

	Rice	Coconut	Maize	Pea
GDOpt	14.9	7.1	91.2	350.1
SimEli-A-RA	7.6	7.5	11.5	514.7
SimEli-HE	-	15.9	78.0	502.3
CH3 E-NE	45.8	37.5	74.3	154.1
CH3 A-NE	74.6	55.7	133.1	86.7
CH3 HE	-	16.6	40.2	62.8

## Discussion

Depending on the purpose of a core collection, a variety of metrics is used to evaluate its quality. Distance-based measures are attractive because they are intuitive to understand and can capture both diversity within the core as well as representativeness of the accessions from the full collection, computed from either genetic markers or phenotypes. However, pairwise distances need to be aggregated in an appropriate way to evaluate the selected core. Although many studies and methods have used average pairwise distance to assess the diversity in the core, it is known that a high average does not guarantee that all accessions in the core are sufficiently different from each other [4, 19]. Maximizing this criterion tends to overrepresent the extremes of the distribution in the full collection.

Core Hunter 2 addressed this issue by maximizing minimum distance in addition to average distance, using a complex mixed replica search (MixRep) consisting of different cooperating strategies [19]. The original Core Hunter software used a local search algorithm to optimize the chosen evaluation measure, but such local searches are not well suited to optimize minimum distance because this measure is very sensitive to the precise selection. Similar cores may have very different values, while at the same time very different cores may have a similar or even the same minimum distance. This makes it difficult for a local search to find its way from a randomly generated selection to a high-quality core. In particular, for a given current solution, many possible modifications may not affect the minimum distance, meaning that the search has no clue as to whether these modifications may eventually lead to an improved solution. To smooth out the objective function, CH2 maximized a combination of average and minimum distance. Also, the applied MixRep algorithm includes a constructive LR heuristic (see “Results”), which is much better suited to maintain a high minimum distance as it iteratively adds accessions to an initially empty selection. Unfortunately, the LR algorithm becomes slow for large datasets, because it builds the core bottom-up, instead of iteratively refining a randomly chosen initial selection.

Two new distance-based metrics, entry-to-nearest-entry (E-NE) and accession-to-nearest-entry (A-NE), introduced by [4], were shown to generate improved cores for specific goals. The E-NE criterion takes all accessions into account and can therefore presumably be more effectively optimized with local searches as compared to minimum distance, but still focuses on maintaining a high distance between each pair of closest accessions which, in contrast to average pairwise distance, avoids overrepresentation of extreme values. Therefore, in Core Hunter 3, the minimum distance measure was replaced with the newly proposed E-NE criterion. The A-NE metric was also included to sample cores that maximally represent all individual accessions from the full collection.

We assessed whether the new E-NE metric can indeed be effectively optimized with local search algorithms, in an attempt to avoid the complexity of the MixRep algorithm used by CH2, and in particular the slowness of the LR replica. We showed that even a very basic stochastic hill-climber (random descent) can already construct cores with high E-NE value and quite little variability in quality across independent samples. Still, the value of the core is further improved, and variability further reduced, when using the more advanced parallel tempering algorithm. Since parallel tempering takes advantage of modern multi-core CPUs, the associated computational overhead is very limited. In our experiments, even for the large pea dataset with over 4000 accessions, parallel tempering was only marginally slower than random descent. We also assessed whether a genetic algorithm could further improve these results. Such global optimization strategy iteratively combines currently known high-quality solutions (crossover) in an attempt to explore other interesting regions of the solution space. The obtained solutions are then exploited by applying local modifications (mutation). We used the random descent heuristic as a mutation operator, since it showed to be able to effectively improve the E-NE value of a given selection. Although the genetic algorithm outperformed random descent, it showed to be slower and produced cores with slightly lower E-NE values as compared to parallel tempering. These results indicate that the intelligent exploitation of parallel tempering is more effective to optimize E-NE than the more global exploration of the evaluated genetic algorithm. We thus conclude that parallel tempering is preferred, and that more complex algorithms are not needed to optimize E-NE, since a basic stochastic hill-climber (random descent) already yields high-quality cores and a global optimization engine (genetic algorithm) did not provide any further advantage. Moreover, parallel tempering does not yield a significant computational overhead—it is almost as fast as random descent. We assume that the same conclusion holds for A-NE due to the very similar composition of both metrics. Therefore, Core Hunter 3 uses parallel tempering by default, which is also known to effectively optimize the other measures that were already included in CH2, such as allelic richness [19]. A fast mode is also provided in which the basic random descent algorithm is applied, in case execution time is critical, but it was not used in this study.

To validate the effectiveness of the new E-NE measure, we assessed whether maximizing E-NE indirectly also yields a high minimum distance. A comparison with Core Hunter 2, configured to sample cores with high average and minimum distance, revealed that this is indeed the case. The minimum distance obtained with CH3 is slightly lower as compared to CH2, but more importantly CH3 yields higher E-NE values because it actively optimizes this criterion. As the minimum distance captures less information about the core than E-NE, we believe that the latter criterion better reflects within-core diversity. As expected, CH3 was faster than CH2 for large datasets, due to the quadratic time complexity of the LR replica. Because of its constructive nature, LR only produces useful results if given enough time to complete. Therefore, a potential additional issue of CH2 is that the user is responsible to set an appropriate time limit that allows the LR replica to complete, when aiming at a high minimum distance. It is not possible to affect the execution time of the LR replica and therefore this method does not provide a quality-runtime tradeoff to the user. Also, it may be confusing that there is a possibly large time gap between the last improvement found by the other replicas and that obtained when the LR replica has finished. In this respect, CH3 is more user-friendly because it uses a well-known local search algorithm that gradually improves the E-NE value of the core. Large gaps between significant improvements are not expected, which makes it easier to determine an appropriate time limit and even more so to use a convenient adaptive stop condition such as a maximum time without finding an improvement, in which case the execution time is automatically adjusted—to some extent—to the size of the collection.

One of the main advantages of Core Hunter 3 and previous versions is its flexibility. While other methods are often developed for a specific purpose such as maximizing diversity, representativeness, or allelic richness, Core Hunter is suited for each of these as it includes a variety of evaluation measures that can directly be optimized, and if desired combined in a weighted index. We compared CH3 with GDOpt, designed to maximize representativeness, and SimEli, where the elimination criterion was chosen either to maximize diversity (SimEli-A-RA) or expected heterozygosity (SimEli-HE). Core Hunter was configured to optimize a weighted index including E-NE and either A-NE (Fig. 2) or HE (Fig. 3), with varying weights, in order to approximate the corresponding Pareto front. The results showed that, as expected, GDOpt is especially suited to construct cores that optimally represent all accessions from the entire collection (low A-NE), as it was specifically developed for this purpose. On the other hand, in terms of diversity (E-NE) and allelic richness (HE), SimEli scores much better than GDOpt. From the two considered elimination criteria, SimEli-HE resulted in the highest allelic richness, while SimEli-A-RA showed to be most suited to maximize diversity (E-NE). Again, this was expected and confirms that the SimEli method can be adjusted to some extent, by using an appropriate elimination criterion depending on the purpose of the core collection. However, Core Hunter 3 found cores that simultaneously have higher E-NE (more diverse), and lower A-NE (more representative) or higher HE values (higher allelic richness), than those obtained by SimEli. In addition, CH3 was able to construct equally representative cores as GDOpt, and thus combines and improves over the advantages of both other methods.

A comparison of execution times showed that CH3 needs less time to optimize HE as compared to E-NE and A-NE. This is not surprising, as it is known that allelic richness can also be effectively maximized with a basic stochastic hill-climber [19]. As we showed that the more advanced parallel tempering algorithm is preferred to optimize E-NE and A-NE, it is clearly more difficult to find cores with high E-NE and low A-NE than to maximize allelic richness. In our experiments CH3 was slower than GDOpt and SimEli for smaller datasets but faster for the large pea dataset. Note that although these methods were implemented in different programming languages, which affects their absolute execution times, the latter does not affect the observed trend in their execution times when sampling from increasingly large collections. Here, the main advantage of Core Hunter is again its flexibility. For example, the runtime of SimEli is determined by the size of the dataset and the sampled core. When sampling a small core from a large collection, many accessions need to be eliminated, and finding the two most similar accessions in each step as well as deciding which one to eliminate requires many computations. In contrast, the runtime of Core Hunter can be adjusted by using an appropriate stop condition. It is possible to limit the total runtime, but we used an adaptive condition that terminated the search when no more improvement was found during 10 s.

There is of course a tradeoff between execution time and solution quality, and we may be able to further increase the quality of the core collections sampled from any of our datasets by allowing a longer runtime. For the large pea dataset for example, we indeed see that allowing no more than 10 s without finding further improvements (Table 2) yields a slightly lower E-NE value as compared to a configuration with an absolute runtime limit of 30 min (Table 1). Since each of the tested methods was able to sample cores from collections with up to multiple thousands of accessions in at most a few minutes, we do not expect that the execution time of any of these algorithms will be limiting for most practical applications. Still, Core Hunter is the only one whose runtime can be controlled by the user in various ways, which yields an interesting quality-runtime tradeoff that can be used to either reduce the execution time for large datasets when needed, or to more thoroughly explore the solution space when more time is available, neither of which is possible with the other methods. Note that although we did not experiment with genotypic datasets with tens or hundreds of thousands of markers, these can easily be dealt with by precomputing a distance matrix, if necessary, so that only the number of accessions affects the performance of Core Hunter.

### Variable size core sampling

Previous versions of Core Hunter also supported variable size core sampling. We decided to remove this functionality from Core Hunter 3, and to focus on fixed size core sampling for the provided evaluation measures, because these measures are not generally applicable to compare cores of different sizes. For example, reducing the core size artificially increases dissimilarity between selected accessions, while adding more accessions always yields a more representative core. Also, while CH1 and CH2 preferred the smallest of two cores with the same objective function value, minimizing the core size may not always be desired, depending on the purpose of the core. We are therefore convinced that fixed and variable size core sampling should be treated as separate problems, using specific evaluation measures and optimization strategies.

## Conclusions

We introduced Core Hunter 3 (CH3) and showed that it constructs core collections with high diversity (high entry-to-nearest-entry distance; E-NE) and which maximally represent the individual accessions from the entire collection (low accession-to-nearest-entry distance; A-NE) using flexible and fast local search algorithms. By default, the parallel tempering algorithm is used. Version 3 improves over Core Hunter 2 (CH2) in multiple ways. CH3 is able to find cores with higher E-NE, within less time for large datasets, which also have a high minimum distance, without the need for a more complex algorithm like the mixed replica search from CH2. In addition, CH3 finds similar and often better cores than GDOpt and SimEli, which were reported to outperform CH2 in terms of E-NE and A-NE. In particular, CH3 can create equally representative cores as GDOpt, which was designed for this purpose, while at the same time being able to construct cores that are simultaneously more diverse, and either are more representative or have a higher allelic richness, than cores obtained with SimEli. As in previous versions, one of the main strengths of Core Hunter is its flexibility. The applied local search algorithms are not confined to a specific evaluation measure and new criteria can easily be introduced and optimized without the need to alter the underlying algorithms. Moreover, multiple criteria can be simultaneously optimized and the execution time is controlled by the user through various stop conditions, which offers a convenient quality-runtime tradeoff. We therefore believe that Core Hunter is a very broadly applicable core subset selection tool with a lot of opportunities to be further extended. For example, we may explore the ability of Core Hunter 3 to sample cores based on a combination of genotypes and phenotypes, or extend Core Hunter to properly incorporate variable size core sampling such as a method to construct covering cores of minimum size.

## Abbreviations

A-NE: Average accession-to-nearest-entry distance
CH2: Core Hunter 2
CH3: Core Hunter 3
DMIN: Minimum distance
E-NE: Average entry-to-nearest-entry distance
GDOpt: Genetic distance optimization
HE: Expected heterozygosity
MixRep: Mixed replica search
REMC: Replica exchange Monte Carlo search
